# Neuroinflammation in glaucoma: a myriad of cellular pathways and players

**DOI:** 10.1007/s00335-026-10253-0

**Published:** 2026-06-27

**Authors:** Michael MacLean, Sean D. Lydon, Cátia Gomes, Elizabeth M. Pizzi, Cory A. Diemler, Sarah E. R. Yablonski, Gareth R. Howell, Jason S. Meyer, Richard T. Libby

**Affiliations:** 1 The Jackson Laboratory, Bar Harbor, ME 04609 USA; 2 Department of Ophthalmology, Flaum Eye Institute, University of Rochester Medical Center, Rochester, NY USA; 3 Neuroscience Graduate Program, University of Rochester Medical Center, Rochester, NY USA; 4 The Center for Visual Sciences, University of Rochester, Rochester, NY USA; 5 Department of Medical and Molecular Genetics, Indiana University School of Medicine, Indianapolis, IN 46202 USA; 6 Stark Neurosciences Research Institute, Indiana University School of Medicine, Indianapolis, IN 46202 USA; 7 School of Graduate Biomedical Sciences, Tufts University School of Medicine, Boston, MA 02111 USA; 8 Graduate School of Biomedical Sciences and Engineering, University of Maine, Orono, ME 04469 USA; 9 Department of Pharmacology and Toxicology, Indiana University School of Medicine, Indianapolis, IN 46202 USA; 10 Department of Ophthalmology, Indiana University School of Medicine, Indianapolis, IN 46202 USA

## Abstract

Glaucoma is a complex neurodegenerative disease with multiple subtypes, yet all are characterized by the progressive dysfunction and loss of retinal ganglion cells (RGCs), which ultimately results in vision impairment and blindness. Elevated intraocular pressure (IOP) is a major risk factor for glaucoma; however, it is neither necessary nor sufficient for glaucomatous neurodegeneration, as patients can exhibit high IOP without developing glaucoma and patients can develop glaucoma with normal IOP. Yet FDA-approved treatment options are largely limited to approaches to minimize risk and reduce IOP. Thus, there is a critical need to target other aspects of glaucoma pathophysiology. Neuroinflammation is broadly defined here as immune-relevant responses, often involving microglia and astrocytes, within the central nervous system which may include peripheral immune cell infiltration. Burgeoning evidence has implicated glia in the development and progression of glaucoma in human tissues and mouse models. Most mouse models of glaucoma to date have shown that microglia and astrocytes are reactive in early stages of glaucomatous neurodegeneration prior to overt RGC loss. However, there is growing evidence that human and mouse glia adopt distinct phenotypes in response to neurodegeneration. Thus, there is critical need to expand our studies to include the new generations of human cell culture models. In this review, we discuss: 1) the evidence of neuroinflammatory processes in human glaucoma; 2) models of glaucoma relevant neuroinflammation; and the evidence specifically for 3) innate immune cell-driven and 4) macroglia-driven processes.

## Introduction

**Fig. 1:**
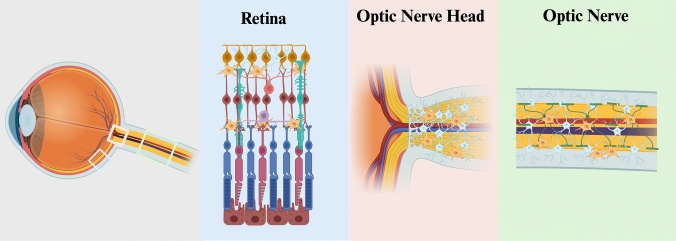
Glaucoma relevant regions and cellular composition. Illustration of retina, optic nerve head, and optic nerve created using BioRender. These three regions are thought to be the most relevant for glaucomatous neurodegeneration. Each tissue displays a unique cellular composition. Astrocytes are light blue, microglia in yellow, Müller glia in turquoise, oligodendrocytes in green, and RGCs are in brown

Glaucoma results in the progressive loss of retinal ganglion cells (RGCs) and vision impairment, which can lead to blindness. Increased age and intraocular pressure (IOP) are major risk factors for developing glaucoma (Quigley 2011). Recent large genome wide association (GWAS) studies have identified hundreds of risk loci (Diaz-Torres et al. 2024; Han et al. 2023). Some of these risk loci are associated with immune-relevant processes and are related to genes highly expressed in glial populations (Hamel et al. 2024; Han et al. 2023). In glaucoma, three distinct CNS regions are of major interest, including the retina, ONH, and ON (Fig. 1). However, there is mounting evidence suggestive that in human glaucoma patients and in mice models there is accompanying brain region specific alterations that mirror retinal RGC loss with disease progression (Hamilton and Kalisch 2025; Cooper et al. 2025; Williams et al. 2013). The impact of glaucoma on the brain was recently reviewed elsewhere (Hamilton and Kalisch 2025). Cell-types commonly implicated in the retina include RGCs, astrocytes, microglia, and Müller glia. The ONH is largely composed of unmyelinated RGC axons, astrocytes, and some microglia. The ON is comprised of myelinated RGC axons, myelinating oligodendrocytes, astrocytes, and microglia. All three regions are vascularized and exhibit blood-CNS-barrier properties. Potential roles for neurovascular dysfunction in glaucoma are reviewed elsewhere (Alarcon-Martinez et al. 2023). Thus, each region has a unique cellular composition and there is burgeoning evidence that cells within each compartment respond to glaucomatous neurodegeneration differently.

Glial cells become perturbed early in pathogenesis, including in the retina, optic nerve head (ONH), and optic nerve (ON) (Mazumder et al. 2023; Amato et al. 2023; Cooper et al. 2016, 2020; Williams et al. 2016; Bosco et al. 2015, 2011, 2008; Howell et al. 2014, 2011; Fan et al. 2010; Zhao et al. 2023; Batsuuri et al. 2025). Thus, these cells are well positioned to mediate early stages of glaucomatous neurodegeneration. However, it is increasingly clear that dampening aspects of neuroinflammation can be beneficial; however, it often can lead to worsened outcomes overall (Cameron et al. 2024; Guttenplan et al. 2020; Liddelow et al. 2017; Bosco et al. 2008; Diemler et al. 2024; Tan et al. 2022). This intriguing duality has been reported for both microglia and astrocytes in glaucoma and underscores potential stage-dependent roles for glia during glaucomatous neurodegeneration. Altogether, these considerations highlight the need to precisely understand the temporal- and spatial- consequences of neuroinflammatory signaling in glaucoma.

Our understanding of the complexities of glial neuroinflammatory responses in central nervous system (CNS) diseases has been rapidly advancing over the last decade. The use of transcriptomics technology, particularly single cell RNA-sequencing (scRNA-seq) has shed considerable light on the context-specific responses of astrocytes and microglia throughout the CNS (Anderson et al. 2022; Hammond et al. 2019; Li et al. 2019c; Keren-Shaul et al. 2017; Cullen et al. 2024; Mazumder et al. 2023, 2022; Liddelow et al. 2017). Studies show that in development, aging, and neurodegenerative diseases of the brain including Alzheimer’s disease (AD), not all glia are actively responding and many appear to be similar to otherwise homeostatic glia in potentially neuroinflammatory contexts (Zhu et al. 2023; Hamagami et al. 2026; Barclay et al. 2024; Krasemann et al. 2017; Keren-Shaul et al. 2017; Hammond et al. 2019). Further, responding glia appear to be in distinct transcriptionally defined states, although how this relates to function is to be determined. These differences likely reflect spatially constricted neurodegenerative phenomena, considering the findings of amyloid plaque-associated microglia both spatially and at the scRNA-seq level (Mallach et al. 2024; Krasemann et al. 2017). Leveraging spatially cognizant analyses in models of glaucoma will help identify causal roles in pathology and identify compartment-specific and cell-state specific phenomena to alleviate pathology.

In this review we highlight evidence of neuroinflammatory processes in human glaucoma; discuss models of glaucoma relevant neuroinflammation; and examine roles for the innate immune system (microglia and peripheral cells) and macroglia (astrocytes and Müller glia) in contributing to glaucomatous neurodegeneration.

## Evidence of Neuroinflammation Driving Human Glaucoma Pathology

There are hundreds of single nucleotide polymorphisms (SNP) that increases the risk for primary open angle glaucoma (POAG), including in genes associated with immune responses (Diaz-Torres et al. 2024; Han et al. 2023). Beyond these risk variants, copy number variations in the gene encoding tumor necrosis factor (TNF) receptor-associated factor NF-κB activator (TANK)—binding kinase 1 (TBK1) have been identified in early onset normotensive glaucoma in multiple populations (Awadalla et al. 2015; Kawase et al. 2012; Fingert et al. 2011). TBK1 has diverse cellular roles including in regulating neuroinflammatory relevant molecules and pathways, including TNF, NF-κB, and interferon (IFN) signaling (Fischer et al. 2025; Ahmad et al. 2016). Several smaller studies have linked SNPs in TNF to POAG (Hamid et al. 2016; Wang et al. 2012). Postmortem investigations of glaucomatous ONH revealed increased TNF expression within microglia and astrocytes relative to controls (Yuan and Neufeld 2001, 2000). Increased TNF and TNF signaling components in postmortem human glaucomatous retinas vs control retinas in multiple studies (Yang et al. 2011; Tezel et al. 2001).

Mutations in *OPTN* encoding optineurin have also been linked to adult onset normotensive POAG (Rezaie et al. 2002). OPTN is known to link autophagy and immune signaling, including regulating NF-κB and IFN signaling (Fukushi et al. 2023). OPTN and TBK1 are known to interact, and their interaction is disrupted by disease-causing variants in TBK1 (Li et al. 2016). SNPs within the adaptor protein TBK1 binding protein 1 have also recently been linked to the development of POAG (Diaz-Torres et al. 2024). Furthermore, SNPs within toll-like receptor (TLR) 4, which is upstream of NF-κB signaling, have been identified in several cohorts of POAG patients but not in others (Navarro-Partida et al. 2017; Mousa et al. 2016; Takano et al. 2012; Chen et al. 2012; Suh et al. 2011; Shibuya et al. 2008). Finally, mutations in myocilin, *MYOC*, are associated with glaucoma and is thought to impact intraocular pressure by effects on the trabecular meshwork (Sharma and Grover 2021). However, recent work has identified myocilin as a unique marker for the specialized astrocytes that compose the *glia limitans superficialis* (which encompasses the surface of the optic nerve and brain) (Hasel et al. 2025). Murine *Myoc* expression has previously been localized to the retinal ganglion cell layer, as well as other ocular regions, but the precise cell type was not clear (Takahashi et al. 1998). It is possible that mutations in *MYOC* could also alter astrocytic function in addition to effects on the trabecular meshwork. Altogether, these studies have provided genetic and postmortem evidence of neuroinflammatory processes in contributing to glaucoma. However, the precise molecular mechanisms underlying these findings or in which cell-types they are acting within is not clear.

Recent work integrating large-scale multi-ancestry GWAS data with single nucleus RNA-sequencing (snRNA-seq) experiments have predicted potential causal genes and cell-types mediating this risk in glaucoma (Hamel et al. 2024). POAG GWAS hits, independent of IOP, were associated with genes highly expressed by glia in the retina, optic nerve, and ONH including astrocytes, Müller glia, and oligodendrocytes (Hamel et al. 2024; Monavarfeshani et al. 2023). Notably, there was no association with microglia. However, this may be due to experimental limitations rather than a true lack of association. It is plausible that this is due to the sparse representation of immune cells in the snRNA-seq dataset and snRNA-seq does not capture important microglial transcripts (Thrupp et al. 2020). Also, the authors were unable to computationally predict causal genes, thus assigning putative cell types to ~ 40% of the GWAS hits (Hamel et al. 2024). It is also possible that these GWAS hits and causal genes could be expressed within a rarer cell-population such as retinal microglia (< 5% of retinal cells) or at lower levels than detectable using standard snRNA-seq approaches. However, it is possible that sc/snRNA-seq technologies could detect rare transcripts, as they have been reported to reach saturation in gene detection at around 1 million reads per cell, which is much higher than in standard experiments at 50–100,000 reads per cell (Svensson et al. 2017). In contrast, the much cheaper bulk RNA-seq approaches could be used to analyze differential alternative splicing and identify rare transcripts with approximately 60 million reads in samples containing purified cell types (Antoszewski et al. 2026). These higher depth approaches may be necessary to more confidently determine whether rare cell types express putative genes and to help predict causal transcripts for some SNPs.

Evidence from postmortem glaucomatous retinal and ONH tissue clearly shows profound morphological changes in microglia, astrocytes and Müller glia relative to control tissues (Salkar et al. 2025; Tezel et al. 2010; Stasi et al. 2006; Yuan and Neufeld 2001, 2000; Pena et al. 1999; Kerr et al. 2011). However, these studies were conducted on postmortem tissues, which can vary in postmortem interval and may not accurately reflect disease progression. One histopathological investigation using enucleated eyes provides support for widespread neuroinflammation in glaucomatous retina and ONH, without the confound of postmortem tissue (Rutigliani et al. 2022). Beyond morphological investigations, components of the innate immune system complement cascade, including C1Q, are increased in glaucomatous retina (Stasi et al. 2006; Tezel et al. 2010). In the ONH, microglia alter the expression levels of the phagocytic marker CD68 and the major histocompatibility complex type II (MHC II) protein HLA-DR, alongside increased TNF levels in both astrocytes and microglia (Yuan and Neufeld 2001). However, the approach to label microglia in these studies has been limited to markers also expressed by peripheral macrophages, and cannot distinguish between the two populations. More refined work will be required to discern if peripheral immune cells are present in the human glaucomatous eye. Connexin 43 (Cx43), an astrocytic gap junction protein, was upregulated in both the human glaucomatous ONH and retina, suggesting glaucoma pathogenesis is associated with altered astrocyte-astrocyte communication (Kerr et al. 2011). Supporting a role for Cx43 in glaucoma pathogenesis, cultured, isolated optic nerve head astrocytes from human tissues exposed to hydrostatic pressure, to mimic increased IOP, showed reductions in gap junctions and altered Cx43 cellular distribution in vitro (Malone et al. 2007).

Collectively, the genetic associations, postmortem evaluations, and the histopathological investigation of donated enucleated tissue strongly indicate that neuroinflammation is not only associated with glaucoma but also likely drives disease progression. The evidence from human studies strongly supports a role for microglia/macrophages and astrocytes in glaucoma. However, it is not clear from these human studies at which stage of disease each cell type actively contributes to pathology and if their role is protective or deleterious.

## Complex Roles for Microglia and Peripheral Cells in the Development and Progression of Glaucoma

### Microglia in Healthy and Glaucomatous Retina

Microglia are resident innate immune cells of the CNS. Within the CNS they are uniquely derived from the embryonic yolk sac rather than neural progenitor cells (Alliot et al. 1999; Ginhoux et al. 2010). In healthy tissue, microglia are important for maintaining synapses and providing neurotrophic support with emerging functions in neurovascular function. Microglia continuously monitor their local microenvironment for molecular signs of distress (Nimmerjahn et al. 2005). Morphologically, this microglial state tends to be ramified with relatively long and complex arbors (Silverman and Wong 2018). In the healthy retina, microglia are often referred to as homeostatic or resting-state microglia, although the precise nomenclature to describe microglia is rapidly evolving. They are most often associated with *Tmem119*, *P2ry12*, and *HexB* expression (Butovsky et al. 2015).

Microglia express a diverse array of pattern recognition receptors, including TLRs. These receptors activate critical pathways that drive the transition from homeostatic to active microglia. Their function and effect on neuronal survival are determined by several factors including disease, tissue, and transcriptional profile (Ebneter et al. 2010). Once activated, homeostatic microglia transition to distinct phenotypes by undergoing morphological and behavioral changes—they become motile, proliferate, and migrate to the site of injury. Morphological changes vary but may include retracting their processes to become ameboid in appearance. Activated microglia can also undergo hyper- or hypo-ramification. The activated microglia population is heterogeneous and cannot be fully described by a single dimension. For a review of the limitations of current microglia nomenclature and a framework for studying microglia, see recent literature (Paolicelli et al. 2022).

### Microglial Transcriptional Diversity

In glaucoma, microglia are generally regarded as the initial responders to axonal injury. For example, in one study using DBA/2J mice, a genetic model of chronic glaucomatous ocular hypertension, microglia were activated in the retina and ONH as early as 3 months – roughly 6 months before RGCs began to die (Bosco et al. 2015, 2011). Some studies show that microglia contribute to glaucomatous neurodegeneration, but others found that microglia play protective roles (Bosco et al. 2008; Diemler et al. 2024; Yang et al. 2013; Breen et al. 2016). Pioneering work using scRNA-seq identified diverse microglial transcriptional states within the brain during neurodegenerative disease, which may perform a wide range of functions (Keren-Shaul et al. 2017; Krasemann et al. 2017). These diverse transcriptional states are context dependent and recent evidence suggests that they may be transient (Hamagami et al. 2026; Barclay et al. 2024). Critically, some activated microglia states have been found to be associated with pathology, aging, and CNS region (Hammond et al. 2019; Keren-Shaul et al. 2017; Krasemann et al. 2017; Anderson et al. 2022; Li et al. 2019c). The most well-studied microglia states are dependent on activation of triggering receptor on myeloid cells 2 (TREM2) – often termed disease associated microglia (DAM) (Krasemann et al. 2017; Keren-Shaul et al. 2017). These are associated with downregulation of *Tmem119*, *P2ry12*, *Cd33* and upregulation of *Lpl*, *Trem2*, *Lgals3*, *Clec7a* and *Apoe*. Functionally, they are associated with phagocytosis of cellular debris and the compaction and clearance of amyloid in Alzheimer’s Disease (AD), and activation of TREM2 in glaucomatous neurodegenerative models is also thought to be associated with these roles. However, concise studies examining microglial states and their roles in models of chronic glaucoma are currently lacking. These studies would enable the detection of these transcriptional states and determine if the same pathways may be functioning in glaucoma as in AD.

### Reactive Microglial States in Glaucomatous Neurodegeneration

Accumulating evidence from human data and mouse models suggests that DAM-like microglia play a crucial role in the pathogenesis of glaucoma. TREM2 is a surface receptor that binds to a broad range of damage associated molecular patterns (DAMPs) – many of which are present early in glaucoma. Recently, data from Chen et al. found that DAM-like microglia are present in patients with glaucoma and that TREM2 activation likely drove expression of that phenotype (Chen et al. 2026). Chen et al. also found that *Trem2* exacerbated RGC death following ischemia–reperfusion injury in mice, although this finding does not appear to translate to the controlled optic nerve crush (CONC) model (Gu et al. 2025). TREM2 activation has also been associated with anti-inflammatory factors such as TGFβ (Li et al. 2019a). Apolipoprotein E (APOE) is a lipid trafficking protein that has been shown to interact with TREM2 to give rise to a DAM phenotype (Krasemann et al. 2017; Yeh et al. 2016; Atagi et al. 2015). The *APOE4* allele is a major risk factor for developing late-onset AD, whereas as *APOE2* is neuroprotective and *APOE3* is neutral (Belloy et al. 2019; Strittmatter et al. 1993; Corder et al. 1993). Investigations into *APOE* isoforms and glaucoma risk have often shown conflicting results but in a recent study *APOE4* was associated with reduced risk for glaucoma (Yi et al. 2024; Margeta et al. 2020). *Apoe* deletion in microbead-induced glaucoma protects against RGC loss and suppresses retinal DAM signatures including *Spp1*, *Lgals3* and *Gpnmb* (Margeta et al. 2022). Additionally, humanized *APOE4* mice are protected against RGC loss in a model of microbead-induced glaucoma and exhibit a suppressed DAM phenotype relative to either *APOE2* or *APOE3* mice (Margeta et al. 2022). This was attributed to microglial-dependent expression of *APOE3* as *APOE3* deletion from *Cx3cr1*^+^ cells was sufficient to protect against RGC loss (Margeta et al. 2022).

scRNA sequencing of retinas from *Optn* E50K mice uncovered a DAM-like microglial population characterized by elevated expression of *Cd74* (Liu et al. 2025). This cluster showed enrichment in MHC-II signaling pathways and expression of other canonical DAM genes, including *Apoe* and *Tnf*. This population could be engaging in MIF-CD74 signaling as migration inhibitory factor (MIF) is a neuronal-derived cytokine that is known to bind CD74 and promote inflammation, including TNF release, which could be contributing to the observed RGC loss (Liu et al. 2025). Separate work also provided evidence of a DAM-like phenotype of CD74^+^ microglia within the ON of silicone-oil-induced glaucomatous mice (Maurya et al. 2024). This CD74^+^ microglia population can be modulated by neuroprotective signaling via Lipoxin B4 secreted by astrocytes (Maurya et al. 2024).

Conversely, in a separate investigation of aged *Optn* E50K retinas revealed a downregulation of type-I interferon signaling pathways and genes including *Ifitm3*, *Oas12* and *Irf7* within microglia (Qiu et al. 2025). Interferon-responding microglia (IRMs) are classified by a type-I interferon signature which has been reported in both humans and mice (Sun et al. 2023; Hammond et al. 2019; Escoubas et al. 2024). IRMs play a critical role in regulating cortical circuitry in developing mouse brains and have been implicated in amyloid plaque responses in mouse models of AD (Roy et al. 2022, 2020; Escoubas et al. 2024). IRF7 is a key transcription factor in the regulation of type-I interferon signaling and is broadly implicated in several different inflammatory pathways. Notably, an interaction between IRF7 and NLRP3 inflammasome has been reported, which may contribute to RGC degeneration (Qiu et al. 2025; Feng and Liu 2016).

One pathway that can activate IFN signaling is the cGAS-STING pathway which is an innate immune system response that detects cytosolic double-stranded DNA (dsDNA) including self-DNA (e.g., mitochondrial DNA) that can be released from cell damage or stress. cGAS (GMP-AMP synthase) functions as a nucleic acid sensor by binding DNA within the cytoplasm and synthesizes the second messenger 2′3’-cyclic GMP-AMP (cGAMP) in response. cGAMP binds to the stimulator of interferon genes (STING) protein which recruits TBK1 to phosphorylate IRF3 thus promoting transcription of interferon-stimulated genes and inflammatory cytokine production (e.g., TNF) (Decout et al. 2021).

Interestingly, the cGAS-STING pathway has also been reported to be activated in mouse models of glaucoma and is present in human patients with end-stage glaucoma (Liu et al. 2024; Wu et al. 2023). In a mouse model of retinal ischemia/reperfusion, dsDNA has known to be released following injury and primarily found to be in the cytosol of retinal microglia (Wu et al. 2023). Additionally, there is an observed time-dependent cGAS-STING elevation following injury, this is associated with microglial activation and RGC damage (Wu et al. 2023). After CONC or microbead induced ocular hypertension, microglia display significant cGAS-STING pathway activation (Liu et al. 2024). Genetic *Sting1* deletion in microglia has been reported to dampen RGC loss following CONC and induce reactivity of retinal macroglia (Liu et al. 2024). The authors created a phosphomimetic *Tbk1* knock-in mouse model *Tbk1*^*S511E/S511E*^ effectively priming TBK1 for activation and thus is hypersensitive to STING signaling. Overactive cGAS-STING-TBK1 enhanced RGC loss while inhibition of TBK1 following CONC showed protection against RGC loss (Liu et al. 2024). Thus, targeting the cGAS-STING-TBK1 pathway and downstream IFN signaling within glaucoma might be a therapeutically beneficial avenue in mitigating glaucoma progression of neuroinflammation and subsequent RGC loss. Targeting specific pathways in glaucoma, such as the cGAS-STING-TBK1 pathway, TREM2, and TNF, could be therapeutically tractable, as other beneficial functions of microglia would be unaffected, such as neuronal/vascular supportive functions. Further, these pathways are all under intense clinical investigation for other diseases including AD and systemic lupus (Mummery et al. 2026; Jaeger et al. 2025; Valencia et al. 2025). If these trials are successful, adoption of these approaches in glaucoma may be warranted after preclinical investigation. Altogether, microglial transcriptional states may distinctly influence RGC outcomes, though whether these effects are protective or deleterious remains unclear.

### Peripheral Infiltration of Macrophages and Monocytes

The complexity of microglial responses may be further modified by direct interactions with the peripheral innate and adaptive immune systems. It is thought that an important contributor to neuroinflammation in glaucoma is the infiltration of macrophages and monocyte-derived cells into the ONH, a critical site of early injury (Williams et al. 2019, 2017a; Howell et al. 2012). In the ONH of DBA/2J mice, monocytes are present prior to RGC loss but are largely absent in normotensive controls (Williams et al. 2019). Multiple studies have manipulated monocyte recruitment to assess the role of these cells in glaucomatous neurodegeneration. Genetic ablation of *Itgam* (CD11b), an adhesion molecule required for monocyte extravasation, reduced glaucomatous neurodegeneration in DBA/2J mice (Williams et al. 2019). Similarly, pharmacological inhibition of platelet–monocyte adhesion using the engineered peptidoglycan DS-SILY, which competitively blocks endothelial surface ligands, decreased immune cell extravasation into the eye and delayed the loss of RGC soma and axons (Williams et al. 2019). Intriguingly, eye-specific x-ray radiation inhibited monocyte entry into the ONH and protected against glaucomatous damage (Howell et al. 2012). In contrast, deletion of *Glycam1*, a negative regulator of leukocyte extravasation, increased monocyte-derived cell infiltration into the ONH of radiation treated mice, however, it only partially abrogated the radiation-induced protection (Williams et al. 2017a). In human disease, patients with normal-tension glaucoma exhibit elevated blood levels of monocyte chemoattractant protein-1, implicating potential activation of peripheral monocyte recruitment pathways in glaucoma pathogenesis (Lee et al. 2017). Further, one postmortem investigation of human glaucomatous optic nerves found increased CD163 + macrophages, which the authors ascribe as peripheral monocyte-derived cells, however, additional work is required to discern if these are truly peripherally derived cells or activated microglia (Margeta et al. 2018; Rutigliani et al. 2022).

Evidence from human postmortem tissue indicates that the adaptive immune system may also be engaged within the retina. Glaucomatous retinas exhibited increased deposition of IgG within the RGC layer, accompanied by CD27⁺/IgG⁺ plasma cells and occasional T lymphocytes, consistent with recruitment of peripheral immune cells into the retina (Gramlich et al. 2013). IgG deposits co-localized with activated microglia and may, at least in part, explain the elevated levels of TNFα, IL1β, IL6, and IL8 detected (Gramlich et al. 2013). In another histopathological study T helper cells, but not cytotoxic T cells, were rarely and inconsistently detected in glaucomatous retina (Salkar et al. 2025). This was accompanied by changes to endothelial tight junction proteins, including Occludin 1 and Claudin 5 (Salkar et al. 2025). Beyond changes to tight junctions, which could allow peripheral immune cell entry, optic disc hemorrhages are known to be associated with significantly increased risk and progression of glaucoma, suggesting that vascular damage could accelerate or cause the direct entry of peripheral cells and adaptive immune molecules, including IgG (Budenz et al. 2006).

Adoptive transfer of purified splenic CD3⁺ T cells, but not CD19^+^ B cells, from glaucomatous donor mice into control healthy mice is sufficient to induce RGC loss, whereas transfer from healthy or immunodeficient glaucomatous donors does not result in RGC loss (Gramlich et al. 2015). Notably, only rare donor lymphocytes are detected within recipient retinas, yet focal microglial activation and lymphocyte–microglia interactions are observed (Gramlich et al. 2015). It is not clear whether the transferred T cells were CD4 or CD8 T cells, or a mixture of both. These data suggest that the observed adaptive immune-mediated neurodegeneration is amplified by resident microglia rather than widespread lymphocytic infiltration, but this was not explicitly tested. Further work is required to understand the precise cellular interactions between the adaptive immune system and microglia. Altogether, these studies support a model in which adaptive immune responses interact with microglia to accelerate RGC degeneration and promote feed-forward inflammatory loops potentially potentiated by vascular damage.

### Extracellular Signaling–Chemokines and Cytokines

Consistently, proinflammatory signaling molecules are upregulated in human patients with glaucoma and in mouse models of glaucoma. Our group and others have found that the complement system is a major contributor to the proinflammatory environment (Hoppe et al. 2025; Soto and Howell 2014). During normal development, the complement system helps regulate neural circuits by tagging unwanted synapses for microglial pruning (Stevens et al. 2007). Similarly, in aged DBA/2J mice the complement system is critical for clearing dying cells and synapses, but can also promote neuronal death with prolonged activation (Williams et al. 2016; Howell et al. 2014, 2013b, 2011). Canonically, activation of the classical pathway through C1Q leads to the formation of the membrane attack complex comprised of C5b, C6, C7, C8, and C9 which binds to target cell membranes to induce lysis and death (Nauta et al. 2004). Interestingly, DBA/2J mice do not express a functional C5 protein (Wetsel et al. 1990), preventing the formation of the membrane attack complex. Instead, C3a acts as a proinflammatory signaling molecule contributing to chronic inflammation through recruitment of microglia and monocytes and suppression of IL10 (Norden et al. 2014; Harder et al. 2020). When a functional C5 gene was backcrossed to DBA/2J mice it exacerbated glaucomatous neurodegeneration and the membrane attack complex was deposited at RGC somas and sites of axonal damage (Howell et al. 2013b). The complement system and its role in eye and brain diseases has recently been reviewed in detail (Daskoulidou et al. 2026).

Coinciding with activation of the complement system is upregulation of other critical inflammatory molecules, namely TNF and IL1α. TNF is a major component of the pro-inflammatory signaling within glaucomatous tissues (Kitaoka et al. 2006; Tezel 2008; Tezel et al. 2001; Hu et al. 2020; Cueva Vargas et al. 2015; Andersh et al. 2026). Besides its role as a strong driver of pro-inflammatory phenotypes in microglia, TNF can affect the sterile alpha and TIR motif containing 1 (SARM1) pathway in RGCs in a neurotoxic manner (Ko et al. 2020). We recently provided evidence that IL1α potentiates the neurotoxic effect of TNF (Andersh et al. 2026). Together, C1Q, IL1α and TNF are thought to drive a neurotoxic astrocyte phenotype that contributes to RGC death (Guttenplan et al. 2020; Sterling et al. 2020; Liddelow et al. 2017). Thus, the innate and adaptive immune systems can promote glaucomatous neurodegeneration by releasing proinflammatory factors including TNF, complement proteins, interleukins, and other cytokines. There is growing appreciation that the consequence of these molecules is not limited to RGCs alone but that they can modify other tissue resident cells including as astrocytes and Müller glia.

## Loss of Protective Functions and/or Deleterious Roles for Macroglia

### Neuroprotective or Neurotoxic–Heterogeneous Functions for Astrocytes in Glaucoma

Astrocytes are thought to be the most abundant glial cell in the CNS, and it is now well appreciated that astrocytes perform a vast array of functions to support neural and vascular processes (Lee et al. 2022). Tremendous work has identified context- dependent responses by astrocytes in health and disease (Zhu et al. 2023; Mazumder et al. 2023, 2022; Cullen et al. 2024; Lee et al. 2022; Liddelow et al. 2017). Regional differences among astrocytes are striking with astrocytes adopting unique region dependent functions (Cullen et al. 2024; Zhu et al. 2023; Mazumder et al. 2023, 2022). Ribotag experiments provided evidence that even within the visual system, astrocytes of the retina, ON, and ONH are molecularly distinct (Cullen et al. 2024; Mazumder et al. 2023, 2022). These regional dependent differences highlight the uncertainty involved with generalizing findings discovered in astrocytes from other CNS regions.

Further, in the face of neural damage astrocytes respond in specific manners depending on the insult (Cameron et al. 2024; Liddelow et al. 2017; Mazumder et al. 2023). Classically, the sole use of increased expression of glial fibrillary acidic protein (GFAP) and morphological changes was used to denote astrocyte reactivity and is increased in postmortem and enucleated glaucomatous eyes relative to controls (Salkar et al. 2025; Rutigliani et al. 2022; Yuan and Neufeld 2000; Pena et al. 1999). Interestingly, increased astrocyte and microglial reactivity in the ON positively correlated with neural damage in DBA/2J mice (Bosco et al. 2016, 2015, 2011). This increased glial activity is observable as a glial “scar” without direct immunostaining for astrocytes or microglia suggesting profound extracellular remodeling of the damaged ON – which has also been reported in human glaucomatous ONH (Bosco et al. 2016; Pena et al. 1999). This remodeling occurs alongside axonal degeneration and occurs without frank astrocytic proliferation (Cooper and Calkins 2024).

A landmark study provided evidence that astrocytes can adopt a neurotoxic “A1” state in response to microglial-derived factors including IL1α, TNF, and C1Q or adopt a neuroprotective state “A2” (Liddelow et al. 2017). Inhibiting microglial secreted factors, either through genetic or pharmacological intervention, significantly reduced RGC loss in response to CONC. Later work from the same group showed that A1 astrocytes produce a toxin that actively kills stressed/damaged RGCs but is insufficient to kill otherwise healthy RGCs (Guttenplan et al. 2020). However, astrocytes can express markers for both A1 and A2 states simultaneously and this binary classification system is likely unable to reflect the true heterogeneity of astrocytic responses (Zhu et al. 2023). Recent work reported two clusters of reactive astrocytes to CONC –separable by their expression of complement component 3 (C3) – which was previously identified as a marker of A1 but not A2 astrocytes (Cameron et al. 2024). Sub-clustering the CONC A1 and A2 clusters revealed further heterogeneity within both populations. CONC induced proliferative C3^−^ astrocytes and enhanced their proliferative capacity, reduced the abundance of C3^+^ neurotoxic astrocytes, possibly by mediating changes in microglia, and led ultimately to reduced RGC death, supporting a new model in which neuroprotective astrocytes are upstream of the microglial-driven neurotoxic astrocytic effects on RGCs. However, CONC is an extreme insult and may not reflect chronic neuroinflammation associated with glaucoma – see above.

There are unresolved differences in reports surrounding the induction of A1 astrocytes in models of microbead-induced ocular hypertension (Guttenplan et al. 2020; Mazumder et al. 2023). Guttenplan et al. report strong induction of A1 transcripts after 30 days of ocular hypertension in the retina, ON, and ONH, which was prevented by global deficiency of *Il1a*, *Tnf*, and *C1q*. However, bulk RNA-seq on ON after 4-weeks of microbead-induced ocular hypertension only found some induction of both A1 and A2 astrocyte signatures, but with no clear preference for either (Zhu et al. 2023). Conversely, Mazumder et al. did not detect induction of A1 or A2 signatures at 7 or 30 days post ocular hypertension in ON or ONH in a ribotag enrichment of astrocyte transcript (Mazumder et al. 2023). However, it is possible that the transcripts associated with A1/A2 astrocytes are sensitive to differences in detection methods utilized and were missed by ribotag enrichment.

Zhu et al. reported an increase in phagocytic genes in ON bulk RNA-seq after 4 weeks of ocular hypertension. The authors confirmed the presence of phagocytic astrocytes in the glaucomatous ON by visualizing axonal-derived mitochondrial material within astrocytes. Changes in phagocytic and proteostasis pathways were confirmed in ribotag-isolated ONH astrocyte transcripts after 30 days of ocular hypertension (Mazumder et al. 2023). Adding to the complexity, ONH and ON astrocytes each displayed unique differential gene expression patterns after ocular hypertension (Mazumder et al. 2023). ON astrocytes exhibited greater differential expression after 7 days compared to ONH astrocytes. Yet, by one month, both populations shared similar total numbers of genes changed and some shared features, including metabolic gene expression rewiring (Mazumder et al. 2023). Many of the earliest transcripts altered in ON astrocytes involved extracellular matrix, cell junction proteins, and cytoskeletal structural proteins, including intermediate filaments, possibly reflecting increased mechanical stress on astrocytes associated with glaucoma (Mazumder et al. 2023).

Interestingly, Mazumder and colleagues reported that they were unable to detect differences in typical neuroinflammatory signatures previously linked to glaucoma in astrocytes and that many of these molecules were found in the input fraction rather than the astrocyte ribosome-enriched fraction (Mazumder et al. 2023). In vitro experiments provided evidence that human pluripotent stem cell (hPSC) derived *OPTN* (E50K) astrocytes exhibit abnormal cellular functions, including increased inflammatory and decreased cell-cycle pathway expression relative to isogenic controls (Gomes et al. 2022). Interestingly, hPSC-derived *OPTN* (E50K) astrocytes induced neurodegenerative phenotypes, including hyperexcitability in co-cultured otherwise healthy RGCs, relative to isogenic control astrocytes (Gomes et al. 2022). RGC health was decreased when treated with OPTN (E50K) astrocyte conditioned media relative to isogenic control astrocyte conditioned media due to loss of protective levels of IL6 (Gomes et al. 2022). These studies provide support for a protective astrocytic response in glaucomatous neurodegeneration.

Given neuroprotective astrocytes have been identified upstream of neurotoxic astrocytes in CONC it is possible that the toxic functions ascribed to astrocytes by Guttenplan and Liddelow et al. depend on the failure of protective astrocytes (Cameron et al. 2024; Liddelow et al. 2017; Guttenplan et al. 2020). Interestingly, many astrocytes are not reactive in the ONH post-IOP increase in the microbead model, suggesting that subpopulations may be responding at any one time (Zhu et al. 2023). Adding to this complexity, astrocytes form interconnected networks that can span large distances within the brain and between the eyes (Cooper et al. 2026, 2020). These networks enable metabolic resources to be shared between affected and unaffected eyes after a microbead-associated increase in IOP, via Cx43 (Cooper et al. 2020). While this process appears protective and reduces glaucomatous neurodegeneration in the injured tissue, it results in contralateral changes that render the contralateral tissue more susceptible to future damage (Cooper et al. 2020). In this vein, inhibition of Cx43 worsens outcomes in the early stages of glaucomatous damage, 4 weeks after an IOP increase in the affected eye (Zhao et al. 2023). However, this seems to shift and by 8 weeks post IOP-increase Cx43 inhibition is protective for RGC loss in the affected eye (Batsuuri et al. 2025). These stage-dependent differences may, in part, be due to inflammatory signals transmitted via gap junctions, but this is not yet clear. scRNA-seq and spatial-based analyses are both required to more accurately identify and localize reactive astrocytes in the retina, ONH, and ON in models of chronic disease. Understanding what other cell-types or structures are adjacent to the reactive cells may unveil new therapeutic targets to bolster protective functions or ameliorate deleterious ones.

### Roles for Müller Glia in the Pathogenesis of Glaucoma

Müller glia are the most abundant macroglial cell within the retina and display a myriad of regulatory functions in health and disease (Arrigo et al. 2025). When Müller glia respond to pathology, these cells dramatically upregulate GFAP similar to astrocytes (Salkar et al. 2025; Arrigo et al. 2025). Activated Müller glia have been noted in postmortem glaucomatous retinas vs controls and this occurs prior to overt RGC loss in mouse models (Salkar et al. 2025; Fernández-Albarral et al. 2022; Hu et al. 2021; Amato et al. 2023). It is thought that Müller glia respond rapidly to changes in the local environment and can release ATP into the environment activating purinergic receptors expressed on RGCs and microglia (Xu et al. 2022; Hu et al. 2021; Xue et al. 2016; Sugiyama et al. 2013). Müller glia derived ATP acting on RGCs may promote cell-death pathways or promote inflammatory cytokine release (e.g., IL6 and TNF) from microglia. There are reports of increased TNF expression in Müller glia from glaucomatous retina and that inhibition of TNF signaling reduces RGC loss (Cueva Vargas et al. 2015). Altogether, these data suggest that Müller glia can amplify and promote feed-forward loops of inflammatory responses by microglia and contribute to RGC death in glaucoma.

Beyond their direct effects on neuroinflammatory processes, Müller glia perform numerous regulatory functions in the healthy retina (Arrigo et al. 2025). For example, the potassium (K^+^) channel Kir4.1is downregulated in Müller glia from glaucomatous retinas (Ji et al. 2012). Decreased Kir4.1 has been linked to altered neural function and reduced K^+^ buffering, disrupting Müller glia function (Abhyankar et al. 2026; Nwaobi et al. 2016; Ji et al. 2012). The evidence from integrating human GWAS data with cell-type specific expression profiles suggests Müller glia may influence the risk of normal tension glaucoma (Hamel et al. 2024). This could be due to changes in their ability to support proper neuronal function or their role in amplifying inflammation. Further work is required to precisely identify which functions are potentially responsible for mediating this change in glaucoma risk ideally to target and restore homeostatic properties or dampen harmful ones.

Burgeoning work has shown that Müller glia exhibit neurogenic potential in some vertebrate species including zebrafish, but not mammalian vertebrates (Hamon et al. 2016). There is growing interest in understanding this process and leveraging this potential as a putative therapeutic for glaucoma and other retinal diseases. Murine Müller glia can be coerced into a neurogenic state via the overexpression of pro-neural transcription factors, which can then differentiate into retinal neurons after injury (Ueki et al. 2015). The precise combination of transcription factors alters the efficacy of Müller glia neurogenic potential (Todd et al. 2022, 2021; Ueki et al. 2015). Interestingly, microglia seem to reduce the neurogenic potential of Müller glia in this reprograming paradigm (Todd et al. 2020). More extensive reviews of Müller glia functions in health and disease can be found elsewhere (Arrigo et al. 2025).

## Glia—More than Glue

The vast majority of human studies of neuroinflammation and glia in glaucoma have largely been associative and lack mechanistic insight. However, when genetic associations with inflammation are coupled with evidence from mouse models, they overwhelmingly indicate that glial populations in the retina, ON, and ONH play active roles in the initiation and progression of glaucomatous neurodegeneration. It is becoming increasingly clear that microglia and astrocytes exhibit stage- and region- dependent roles in disease. Initial protective responses to neural stress and damage may be protective, however, chronic activation likely results in inflammatory amplification and deleterious outcomes. This is exemplified by studies highlighting the nuances of microglial and astrocyte function in glaucoma models. It is not clear when glia lose their protective functions or if this occurs in spatially restricted regions. Identifying the mechanisms behind this loss of support/gain of toxicity is essential. Thus, future work rigorously dissecting the temporal and spatial roles of glia in glaucomatous neurodegeneration is required. Identifying the right time and place to promote beneficial glial functions and prevent or slow feed-forward inflammatory processes is essential to improve patient outcomes.

## Shared Mechanisms of Glaucoma, Age-Related Macular Degeneration, and Diabetic Retinopathy and the Role of Neuroinflammation

The most common eye diseases worldwide include glaucoma, age-related macular degeneration (AMD), and diabetic retinopathy (DR) (Tham et al. 2014; Wong et al. 2014; Yau et al. 2012). There are several known shared risk factors between all three retinal disorders including diabetes, age, and obesity (Fujita et al. 2023; Yau et al. 2012; Chen et al. 2014; Hsiung et al. 2026; Zhang et al. 2016a). Recent work has identified shared genetic risk factors across all three conditions, including *CDKN2B-AS1* variants (Bai et al. 2026). Variants in *CDKN2B-AS1* have been linked to increased risk of POAG and type 2 diabetes for many years (Burdon et al. 2011; Scott et al. 2007). Recently, *CDKN2B-AS1* variants were linked to advanced stage proliferative DR specifically (Yao et al. 2025). *CDKN2B-AS1* is a lncRNA that has been reported to regulate senesce, inflammation, and metabolism (Li et al. 2019b; Rankin et al. 2019; Gao and Jakobs 2016). However, studies have been limited as the equivalent murine region is not entirely clear. Detecting the proposed murine ortholog *Gm12610* has not been successful, at least in the peripheral tissues examined (Rankin et al. 2019; Holdt et al. 2016). Yet, deletion of 70 kb of the orthologous region (including *Gm12610*) increased susceptibility to glaucomatous neurodegeneration concomitant with apparent increased microglial activation (Gao and Jakobs 2016). Altogether, these considerations suggest that further investigation into the role of *CDKN2B-AS1* on neuroinflammatory processes in ocular diseases is warranted.

Neuroinflammation has been well documented in models of all three diseases including microglial and astrocytic activation. Microglia are also thought to contribute to the pathogenesis of DR and AMD (Yu et al. 2024; Altmann and Schmidt 2018). TNF and the complement cascade have also been implicated in all three disorders suggesting that a common feature of retinal neurodegeneration may include the activation of these pathways (Yednock et al. 2022; Shahulhameed et al. 2020; Howell et al. 2013b; Tezel et al. 2010; Stasi et al. 2006; Gerl et al. 2002).

Beyond similarities in proinflammatory cytokine production, there is also shared mitochondrial functional abnormalities resulting in increased oxidative stress (Mazumder et al. 2023; Tezel et al. 2010; Levkovitch–Verbin et al. 2000; Ferrington et al. 2020). Emerging work has highlighted that proper mitochondrial and metabolic function is essential for microglial responses in models of neurodegeneration (Espinoza et al. 2025; Stoolman et al. 2024; Baik et al. 2019). Further, microglia and astrocytes can donate functional mitochondria to neurons in neurodegenerative contexts (Scheiblich et al. 2024; Hayakawa et al. 2016). These phenomena may be occurring alongside the astrocyte-driven uptake and degradation of RGC axonal mitochondrial material reported in the glaucomatous ONH (Davis et al. 2014; Zhu et al. 2023). When damaged mitochondria are not cleared properly, it can result in the extracellular release of mitochondrial DNA, which in turn promotes activation of the cGAS-STING pathway in microglia promoting neuroinflammation (Miao et al. 2025). Importantly, the cGAS-STING pathway has been implicated in DR, AMD, and glaucoma (Liu et al. 2024; Wu et al. 2023; Hu et al. 2023). Thus, neuroinflammation is the most prominent link between DR, AMD, and glaucoma likely driving retinal neurodegeneration across diseases. It is imperative that we model the most relevant aspects of neuroinflammation to improve translatability. Finally, we discuss some of the models that have been used to investigate the role of neuroinflammation in glaucoma.

## Under Pressure—Modeling Glaucoma Relevant Neuroinflammation In Vivo

**Fig. 2:**
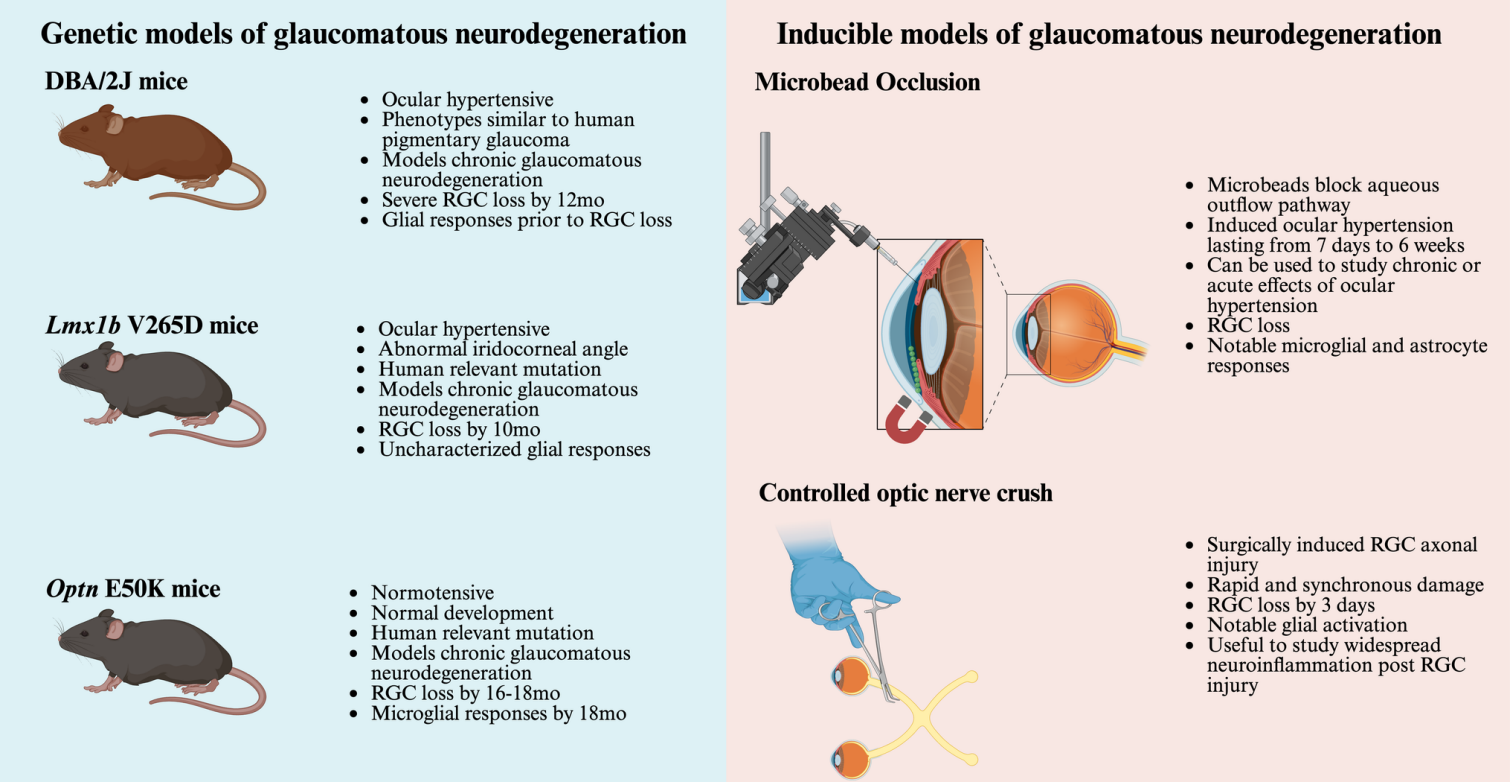
In Vivo Models of glaucomatous neurodegeneration. Illustration of inducible and genetic murine models of glaucoma created using BioRender. Genetic models covered include ocular hypertensive DBA/2J and *Lmx1b* V265D mice alongside normotensive *Optn* E50K mice. All three models are useful to study chronic glaucomatous neurodegeneration and neuroinflammation. Inducible models covered include the widely used microbead occlusion and the controlled optic nerve crush (CONC) model. Microbead occlusion of the aqueous outflow results in ocular hypertension that can be used to study acute or chronic glaucoma and neuroinflammation. CONC results in profound, rapid, and synchronous RGC axonal injury with widespread neuroinflammation. Together, these powerful in vivo models allow for the investigation of genetic risk factors, acute or chronic glaucomatous neurodegeneration and neuroinflammation, and to test therapeutic approaches

It is imperative that we model the most relevant aspects of neuroinflammation to improve translatability. Therefore, we discuss some of the most widely used models to investigate the role of neuroinflammation in glaucoma. First, we discuss murine models of glaucoma, then discuss emerging human induced pluripotent stem cell (iPSC) models (Fig. 2). We focus on murine models, as many of the studies conducted on the role of neuroinflammation in glaucomatous neurodegeneration have been completed using mouse models. However, it is important to note that other rodent models of glaucoma are available (Morrison et al. 2025, 2008; Smedowski et al. 2014). Further, there are also non-human primate models of glaucoma (Wilsey et al. 2016; Burgoyne 2015; Dawson et al. 1993). Comprehensive reviews of models of glaucoma can be found elsewhere (Fernandes et al. 2015; Pang and Clark 2020; Johnson and Tomarev 2010).

## Genetic Models of Chronic Glaucomatous Neurodegeneration

To model the progressive pathology of glaucoma several genetic models of glaucoma have been developed and characterized. These models have been critical for identifying the earliest events in glaucomatous neurodegeneration. Further, these genetic models allow for examining how additional risk factors (diet, genetics, etc.) influence the disease course. These models inherently include an aging component that is not captured by inducible models or by cell culture-based models. Aging is known to influence retinal function and contributes to glial changes independent of disease (Hammond et al. 2019; Marola et al. 2025). Recent work by us and others has shown that genetic context influences retinal aging and the development of glaucomatous neurodegeneration (Anderson et al. 2006; Marola et al. 2025; Tolman et al. 2021).

### DBA/2J Mice

DBA/2J mice develop age-related, asynchronous ocular hypertension. Elevations in IOP begin around 8 months of age and persist until 12–13 months of age (Libby et al. 2005a). This elevation in IOP is the result of iris disease including iris stromal atrophy and iris pigment dispersion (John et al. 1998). Iris stromal atrophy is the result of a mutation in the *Tyrp1* gene (*Tryp1*^*b*^) and iris pigment dispersion results from mutations in the *Gpnmb* gene (*Gpnmb*^*R150X*^) (Anderson et al. 2002; Chang et al. 1999). These phenotypes are similar to those observed in human pigmentary glaucoma (Anderson et al. 2002; Libby et al. 2005a). Corneal calcifications can make IOP measurements difficult in this strain (Turner et al. 2017). Elevations in IOP lead to ON damage and RGC death. By 12 months of age, approximately 70% of ONs are severely damaged (Libby et al. 2005a). RGC death occurs via apoptosis in a wedge-like pattern and is asymmetric between the two eyes (Schlamp et al. 2006; Jakobs et al. 2005; Libby et al. 2005b). Further, RGC death is dependent on IOP elevation, as manipulations that reduce IOP can prevent the glaucomatous neurodegeneration in the retina (Anderson et al. 2006; Matsubara et al. 2006; Schuettauf et al. 2002; Wong and Brown 2012, 2013). Importantly, the DBA/2J disease process includes elements of neuroinflammation including blood retinal barrier breakdown and leukocyte infiltration (Mo et al. 2003; Howell et al. 2012; Williams et al. 2019), glial activation (Inman and Horner 2007; Bosco et al. 2011; Cooper et al. 2016), and complement activation (Fan et al. 2010; Harder et al. 2020; Stasi et al. 2006). However, it is important to note that DBA/2J mice can exhibit auditory seizures and can develop a number of other health concerns that require proper planning to ensure experiments are powered in the face of unavoidable attrition (Turner et al. 2017; Tan et al. 2008). In particular, the DBA/2J model is well-suited to studying chronic neuroinflammatory changes in the glaucomatous disease process.

### B6.*Lmx1b* V265D Mice

Mutations in LIM-homeodomain transcription factor 1 beta (LMX1B) cause nail-patella syndrome (NPS) (Milla et al. 2007). Approximately 29% of patients with NPS over 40 years of age develop ocular hypertension and/or glaucoma (Milla et al. 2007). In both humans and mice LMX1B mutations have incomplete penetrance of eye abnormalities (Cross et al. 2014). *Lmx1b* mutation can lead to diverse effects on irideocorneal angle (Cross et al. 2014; Tolman et al. 2021). By 10 months of age these mice develop severe ON damage with high penetrance, and some mice develop high IOP (Cross et al. 2014; Tolman et al. 2021). When the heterozygous mutation was backcrossed to other mouse genetic backgrounds it became clear that genetic context influenced the severity of the glaucoma-relevant phenotypes (Tolman et al. 2021). 129 mice were resistant to the glaucomatous neurodegeneration associated with the *Lmx1b* mutation (Tolman et al. 2021). Like DBA/2J mice, this model is potentially well-suited for studying the chronic pathologies associated with glaucoma. Plus, *Lmx1b* mutations cause glaucoma-relevant phenotypes in C57BL/6J mice, a common mouse strain. It will be important to characterize the glaucoma-relevant neuroinflammation to enhance its clinical relevance.

### OPTN E50K Mice

Mutations in *OPTN* cause normotensive glaucoma in mice and in humans (Liu et al. 2025; Chi et al. 2010; Rezaie et al. 2002). Mice expressing a disease-associated variant of *OPTN*, *OPTN* E50K, have been generated. The first transgenic overexpression mouse model created exhibited retinal degeneration by 16 months (Chi et al. 2010). More recently, a transgenic mouse model expressing human *OPTN* E50K at approximately physiological levels was generated with seemingly normal retinal development (Tseng et al. 2015). This humanized model exhibits RGC functional decline, damage, and cellular loss by 18 months (Tseng et al. 2015). A CRISPR-Cas9 targeted approach was used to mutate the mouse *Optn* gene to express mutant OPTN E50K (Zhang et al. 2021). Regardless of the OPTN mouse model used, they all appear to be characterized by visual function decline and RGC loss by 18 months of age. Recent work has shown neuroinflammatory processes are active during the window of RGC loss in *Optn* E50K mice by single cell sequencing. But it is not yet clear if these processes are a consequence of neuronal death or actively contribute to the progression of pathology (Qiu et al. 2025; Liu et al. 2025). OPTN mutant mice are an excellent model to probe the neuroinflammatory response in glaucoma in a normotensive environment.

## Experimentally Induced Models of Glaucomatous Neurodegeneration

Various models have been developed to mimic glaucomatous neurodegeneration mechanically. These models include both induced ocular hypertension models (silicone oil, microbead, and hyaluronic acid glycidyl methacrylate) and axonal damage models (controlled optic nerve crush and axotomy) (Pang and Clark 2020; Zhang et al. 2019; Guo et al. 2018; Duan et al. 2015). These models are often considered more acute with timeframes of peak RGC damage and death ranging from days to weeks. It is important to note that in the induced ocular hypertension models, the observed IOP increase can be variable across approaches and if IOP increases are too high it can result in ocular ischemia and confound experiments. These models allow for temporal control of when IOP increases occur, enabling stage specific examination and therapeutic intervention. In this review, we specifically discuss the commonly used ocular hypertensive microbead model and the controlled optic nerve crush model (Fig. 2).

### Microbead Occlusion Model

IOP can be elevated in mouse models via obstruction of aqueous humor outflow. This can be achieved via injection of microbeads that block the conventional outflow pathway. Original work used polystyrene beads, though more recent work has introduced magnetic microbeads, which can be manually guided into the iridocorneal angle (Sappington et al. 2010; Cone et al. 2010; Ito et al. 2016; Chen et al. 2011). The resulting IOP elevation can last from 7 days up to 6 weeks, depending on experimental conditions (Cone et al. 2012; Ito et al. 2016). This is followed by glaucomatous neurodegeneration (Ito et al. 2016; Cone et al. 2010, 2012; Chen et al. 2011; Sappington et al. 2010; Frankfort et al. 2013). The increase in IOP (roughly 30–38%) is similar to that observed in mild/moderate ocular hypertension in human patient eyes (Sappington et al. 2010). Retinal glial cell activation has been noted following microbead-induced ocular hypertension, including in microglia (Kumar et al. 2023; Wang et al. 2025) and astrocytes (Tan et al. 2022; Guttenplan et al. 2020). Additionally, increased complement expression in the retina has been detected following magnetic microbead injection into the anterior chamber (Hoppe et al. 2025; Krishnan et al. 2019). Microglia depletion experiments highlight the importance of microglia in glaucoma (Tan et al. 2022). The microbead occlusion model can be suited to studying either acute or chronic neuroinflammation following induced ocular hypertension.

### Controlled Optic Nerve Crush

Controlled optic nerve crush (CONC) is a mechanical injury to the ON in mice designed replicate the damage to RGC axons observed following elevations in IOP (Whitmore et al. 2005; Quigley and Anderson 1977; Radius and Anderson 1981; Howell et al. 2013a). The ON of one eye is crushed using a pair of self-closing forceps for a short period of time (Li et al. 1999; Levkovitch–Verbin et al. 2000). The exact positioning of the crush site is frequently close to the eye with 1–2 mm behind the globe being common. This procedure results in rapid, synchronous RGC death, with RGC dropout being detectable by three days post-CONC and almost all RGC loss occurring within three weeks of the procedure (Harder et al. 2012; Li et al. 1999; Tsuda et al. 2016). CONC leads to activation of microglia (Gu et al. 2025; Heuss et al. 2018; Mac Nair et al. 2016), macroglia (Sun et al. 2017; Batsuuri et al. 2025; Chucair-Elliott et al. 2022), and infiltration of leukocytes (Cheng et al. 2025; Ha et al. 2017; Kurimoto et al. 2013). The contralateral eye has historically served as a control eye and received a ‘sham’ procedure (Li et al. 1999). Death of RGCs in the sham eye is not commonly observed (Schoot Uiterkamp et al. 2025; Harder et al. 2012). Yet, activation of glial cells has been noted in the sham retina following CONC (Schoot Uiterkamp et al. 2025; Wurl et al. 2023; Cabrera-Maqueda et al. 2023). It is important to note that this injury does not replicate core features of the glaucomatous disease process such as elevated IOP or chronic inflammation. However, the synchronicity and strength of the injury caused by CONC make CONC a useful model for studying widespread neuroinflammatory changes following RGC axonal insult. We next briefly discuss the emerging human induced pluripotent stem cell models and how they have been leveraged to study glaucoma. Additional rodent models of glaucoma are available and have been extensively reviewed elsewhere (Fernandes et al. 2015; Pang and Clark 2020; Johnson and Tomarev 2010).

## Improving Translatability by Incorporating Glaucoma-Relevant Neuroinflammation in Human Cell Models

**Fig. 3:**
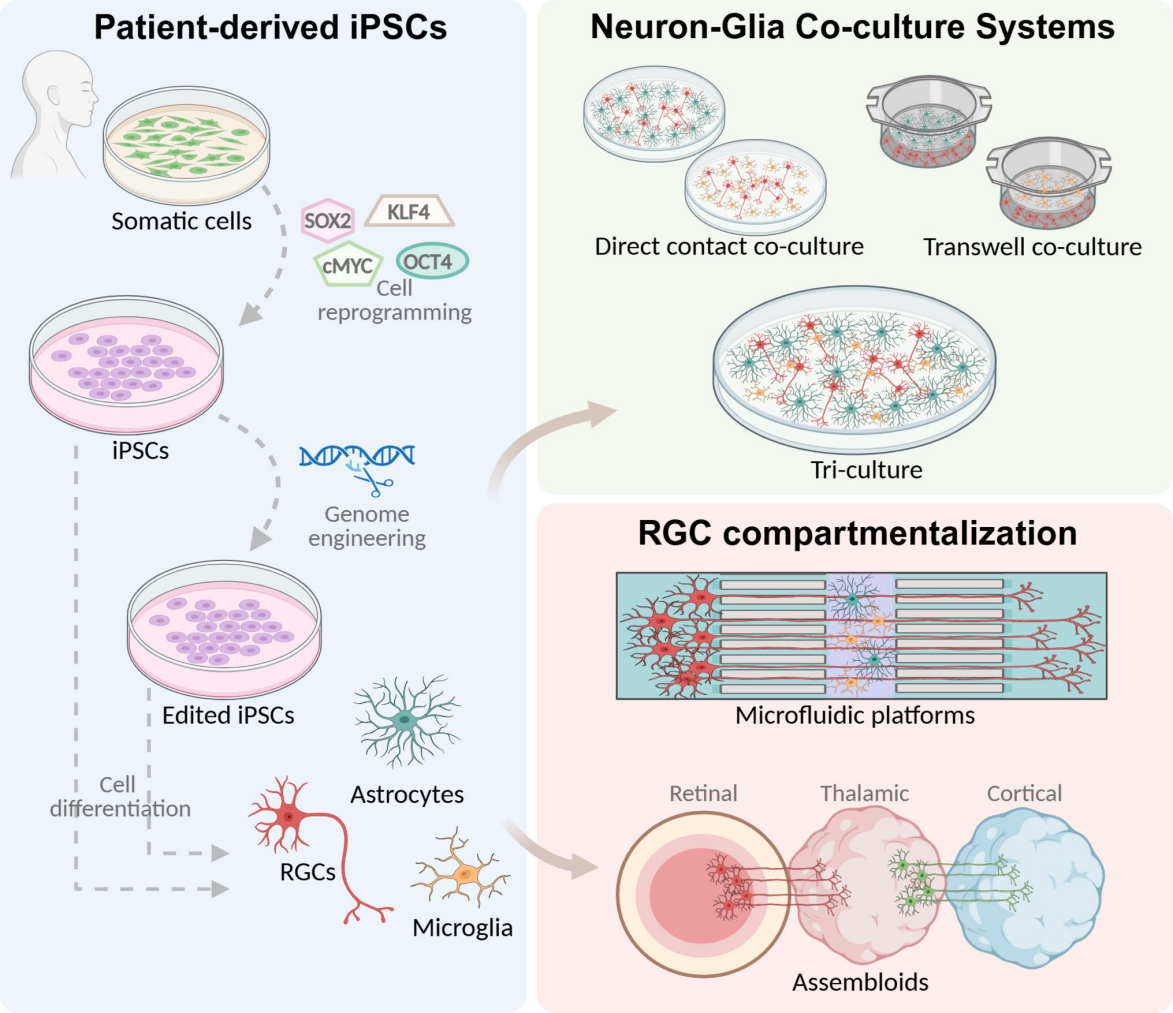
iPSC-based platforms to model glaucomatous degeneration. Illustration created using BioRender. Patient-derived somatic cells can be reprogrammed into induced pluripotent stem cells (iPSCs) through expression of reprogramming factors (e.g., SOX2, KLF4, cMYC, OCT4). Genome engineering enables the generation of edited or isogenic iPSC lines for disease modeling. Differentiation of iPSCs into retinal ganglion cells (RGCs), astrocytes, and microglia, which can be combined in various in vitro systems to model glaucoma-relevant mechanisms. Neuron–glia interactions can be studied using direct contact co-cultures, transwell systems, or tri-culture platforms incorporating multiple cell types. Microfluidic devices enable compartmentalization of RGC somatodendritic and axonal domains, allowing investigation of axon-specific degeneration and localized glial effects. More advanced 3D models, including retinal–thalamic–cortical assembloids, support connectivity and provide platforms to study multicellular interactions within a tissue-like architecture. Together, these human-based systems constitute powerful platforms investigate genetic risk, neuroinflammation, and compartment-specific vulnerability in glaucoma

Experimental models have advanced our understanding of RGC degeneration and glial contributions in glaucoma. However, species-specific differences can limit their translational relevance (Zhang et al. 2016b; Peng et al. 2019). Therefore, human pluripotent stem cells (hPSCs), including patient-derived iPSCs, provide a complimentary, physiologically relevant in vitro human platform to model retinal development, RGC degeneration, and RGC-glia interactions in glaucoma (Fig. 3). Further, these platforms can be used to determine the mechanistic impacts of causal genes on relevant cell type specific cellular functions.

### 3D Retinal Organoids and RGC Differentiation

The limited availability of primary human RGCs has driven the use of hPSCs to generate retinal cells for disease modeling. Importantly, patient-derived hPSCs carrying glaucoma-associated mutations, combined with CRISPR/Cas9 genome editing methodologies, enable the generation of isogenic controls and precisely engineered glaucoma models (Cong et al. 2013; Muffat et al. 2016; Vanderwall et al. 2020). Stepwise differentiation approaches have enabled the generation of 3D retinal organoids that recapitulate human retinogenesis—producing all major retinal cell types (Capowski et al. 2019; Fligor et al. 2018; Nakano et al. 2012; Wahlin et al. 2017; Zhong et al. 2014; Meyer et al. 2011; Harkin et al. 2024). Interestingly, retinal organoids differentiated from POAG patient-derived hPSCs have revealed RGC-specific transcriptional alterations (Daniszewski et al. 2022), and those differentiated from hPSCs carrying the OPTN(E50K) mutation exhibited profound LC3 accumulation exclusively within the inner RGC layers (Vanderwall et al. 2020).

Although 3D retinal organoids recapitulate tissue architecture and multicellular interactions, their complexity can limit analysis of cell-autonomous mechanisms. Thus, most studies use purified RGCs in adherent 2D cultures, which provide a controlled, reproducible and scalable platform for modeling glaucoma-associated neurodegeneration (Huang et al. 2023). RGCs can be isolated from retinal organoids or generated through adherent and inducible transcription factor-based protocols (Vanderwall et al. 2020, 2019; Risner et al. 2021; Sluch et al. 2017; Wang et al. 2020; Agarwal et al. 2023; Liou et al. 2023). These systems enable assessment of neurite outgrowth, molecular pathway alterations, and electrophysiological function, capturing early degenerative changes and functional deficits (Vanderwall et al. 2020; Huang et al. 2024; Surma et al. 2023; Gomes et al. 2022). Importantly, patient-specific iPSC-derived RGCs carrying glaucoma-associated variants, including OPTN(E50K), TBK1 duplications, *MYOC* mutations, and other risk alleles, further enable mechanistic interrogation of disease pathways (Teotia et al. 2017; Tucker et al. 2014; Vanderwall et al. 2020; Shil et al. 2026). Combined with isogenic controls, these models recapitulate key phenotypes such as impaired neurite outgrowth, axonal dysfunction, stress susceptibility, and reduced survival. Notably, OPTN(E50K) RGCs exhibit deficit autophagy and reduced mTOR signaling (Vanderwall et al. 2020; Surma et al. 2023; Huang et al. 2024), linking genetic risk to disrupted cellular homeostasis.

### iPSC Models to Study Glaucomatous Neuron-Glia Interactions

Astrocytes and microglia closely associated with RGCs within the nerve fiber layer of the retina and optic nerve, where they support RGC homeostasis and function. However, during injury or disease, these glial cells acquire a reactive and pro-inflammatory state contributing to RGC dysfunction and degeneration (Morgan 2000; Soto and Howell 2014). Because access to human tissue is limited, in vitro hPSC-based models have become essential for studying neuron-glia interactions. hPSC-derived astrocytes (Krencik and Zhang 2011) have been shown to enhance the maturation and functional properties of hPSC-derived RGCs (Vanderwall et al. 2019), highlighting their supportive role. Importantly, disease modeling has revealed that astrocytes carrying the glaucoma-associated OPTN(E50K) mutation exhibit autophagy dysfunction and inflammatory transcriptional changes and promote degenerative phenotypes over RGCs in co-culture systems (Gomes et al. 2022). Although some glaucoma cases are linked to defined genetic mutations, the majority are considered sporadic, highlighting the need for in vitro models that recapitulate neuron-glia interaction in those patients. Notably, increased glial reactivity and neuroinflammation are broadly observed across glaucoma patients, independent of genetic etiology (Salkar et al. 2024, 2025). In accordance, experimental protocols have been established to induce a reactive phenotype in astrocytes, initially described in rodent cells by Liddelow et al. (2017), and recently adapted for iPSC-derived astrocytes (Gomes et al. 2024; Barbar et al. 2020). Incubation of iPSC-derived astrocytes with TNFα, IL-1α and C1q drives these glial cells toward a reactive pro-inflammatory state (Gomes et al. 2024; Barbar et al. 2020). When co-cultured with RGCs, these reactive astrocytes promote morphological and functional alterations over RGCs, consistent with neurodegeneration (Gomes et al. 2024). Similarly, studies have shown the presence of activated microglia in the ONH of glaucoma eyes (Yuan and Neufeld 2001). Although limited, human in vitro studies have begun to focus on microglial contributions to RGC degeneration. Established protocols enable differentiation of hPSCs into microglia (Mcquade et al. 2018; Abud et al. 2017), which have been widely used to model neuroinflammation and neurodegeneration (Bedford et al. 2025; Tutrow et al. 2025; Haskell et al. 2026). Lipopolysaccharide (LPS) is commonly used to induce a pro-inflammatory microglial phenotype in vitro (Fricker et al. 2012; Cunha et al. 2016), including to model glaucoma (Williams et al. 2017b; Larsen et al. 2025). Indeed, LPS-treated hPSC-derived microglia exhibit increased MHC-II expression, enhanced phagocytosis, and exacerbated secretion of toxic factors (Harkin et al. 2025). Importantly, while non-stimulated hPSC-derived microglia showed a beneficial effect over RGC homeostasis, LPS-activated microglia led to a degenerative phenotype in RGCs, including morphological and functional deficits (Harkin et al. 2025).

Increasing evidence has shown that microglia and astrocytes work together to promote neuroinflammation and RGC degeneration. Accordingly, tri-culture systems containing hPSC-derived neurons, astrocytes and microglia have emerged as powerful platforms to model glial crosstalk and its impact on neurons (Lish et al. 2025). On these platforms, chronic microglia activation induced by LPS promoted astrocyte reactivity and, together, sustained a pro-inflammatory environment that progressively exacerbated RGC degeneration (Harkin et al. 2025). In parallel, 3D retinal organoids incorporating iPSC-derived microglia have been developed to better recapitulate tissue architecture (Chen et al. 2025; Chichagova et al. 2023). Integrated microglia remain functionally responsive and adopt pro-inflammatory phenotypes following LPS exposure (Chichagova et al. 2023). Notably, these findings are complimented by the observation that iPSC-derived macrophage precursor cells can infiltrate 3D retinal organoids and differentiate in situ into microglia-like cells (Usui-Ouchi et al. 2023). While these systems provide promising platforms to study neuroinflammation in a tissue-like context, further studies are needed to determine whether they fully recapitulate key features of glaucomatous neurodegeneration.

### Modeling RGC Compartmentalization

RGCs are highly polarized and compartmentalized neurons, with distinct somatodendritic and axonal domains that support specialized functions, with the axons being particularly vulnerable in ON injury and glaucoma (Syc-Mazurek and Libby 2019; Donato et al. 2019). However, hPSC-derived RGCs often exhibit immature morphology, limiting in vitro modeling of compartment-specific mechanisms. Microfluidic devices, capable of isolating axonal compartments of neurons (Taylor and Jeon 2011), have emerged as powerful tools to enhance RGC polarization and enable selective manipulation of neuronal compartments (Fligor et al. 2021; Teotia et al. 2019; Boal et al. 2023; Gomes et al. 2025). Using these systems, selective axonal injury recapitulates key glaucomatous features, including impaired anterograde transport, axonal varicosities, and progressive degeneration (Boal et al. 2023). In addition, OPTN(E50K) mutant RGCs exhibited impaired axonal outgrowth and reduced axonal transport efficiency, with axonal RNA sequencing highlighting disease-associated transcriptomic alterations specific to glaucomatous RGC axons (Gomes et al. 2025). Together, microfluidic platforms provide a translationally relevant approach to study axon-specific mechanisms underlying glaucomatous degeneration. Taking into account that early glaucomatous axonal injury correlates with focal glial reactivity at the ONH (Mac Nair and Nickells 2015), three-chamber microfluidic devices provide a powerful platform for modeling compartment-specific neuron-glia interactions. In this system, astrocytes positioned along the proximal compartment induce localized degenerative changes in RGCs axons when driven to a reactive state, demonstrating axon-specific vulnerability (Gomes et al. 2025). Incorporating additional cell types, such as microglia and vascular elements, will further enhance physiological relevance.

Beyond microfluidic systems, 3D models of the human visual system provide complementary platforms to study RGC biology and degeneration in disease. Assembloids integrating retinal, thalamic and cortical organoids enable RGC axon extension toward brain targets and improve long-term survival (Fligor et al. 2021). Adding glial populations to these platforms represents a promising next step for modeling neuroinflammatory mechanisms underlying glaucomatous degeneration in a more physiologically relevant multicellular context.

It is imperative that, as the field moves forward, the research is anchored in the factors driving human disease. Incorporating murine glaucoma models alongside human cellular models to interrogate specific molecular pathways driving glaucoma-relevant neuroinflammation represents a yet untapped opportunity for therapeutic intervention. Further, it is plausible these platforms could be used to test causal genes and cell-types identified in the large GWAS studies to pinpoint the mechanistic impact on intrinsic cellular functions before testing the role of the gene in murine models of glaucoma.

## Conclusion

**Fig. 4:**
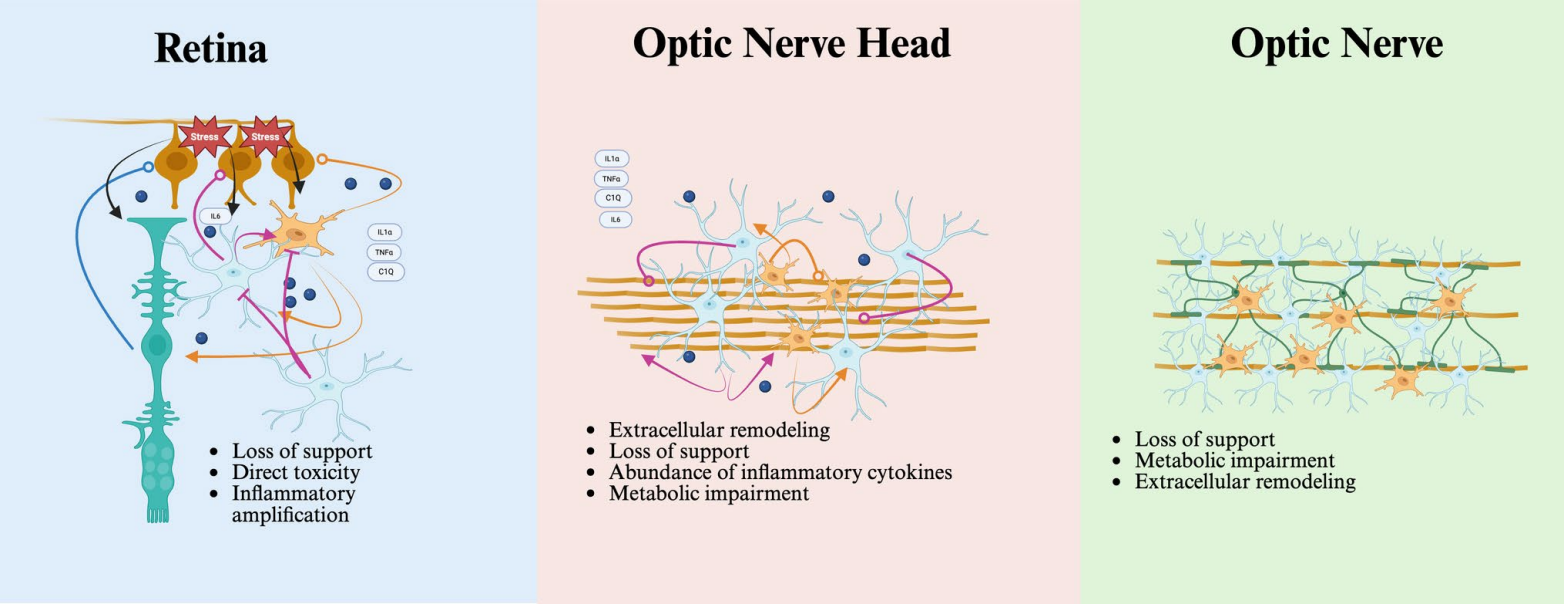
Glaucoma relevant regions and cellular composition. Illustration of retina, optic nerve head, and optic nerve created using BioRender. Within each region there are complex cellular-communication pathways active that both inhibit and promote RGC stress. Further, glia can actively promote neurotoxic or neuroprotective phenotypes in other glia in addition to having direct effects on RGCs. Astrocytes are light blue, microglia in yellow, Müller glia in turquoise, oligodendrocytes in green, and RGCs are in brown. Open circles represent potential neurotoxic and/or loss of neurosupportive pathways, lines represent inhibitory pathways, and arrows reflect inflammatory amplification signaling

It is increasingly clear that the progression of glaucomatous neurodegeneration is influenced by neuroinflammatory processes driven by glial cells in the retina, myelinated optic nerve, and unmyelinated optic nerve head. These processes are complex and are both protective and damaging (Fig. 4). The overwhelming heterogeneity of glial cells underscores the requirement for spatially and temporally conscious analyses to identify specific glial populations and cell–cell communication pathways active in regions most at risk for damage and degeneration. These future experiments are necessary to develop targeted therapies to sway the balance between harmful and protective neuroinflammatory pathways.

## Data Availability

No datasets were generated or analysed during the current study.
